# Feud, Flower, and Fatal Electrocardiograms

**DOI:** 10.7759/cureus.52531

**Published:** 2024-01-18

**Authors:** Sriram Veeraraghavan, Bharath Raj Kidambi, Sai Krishna Reddy, Soorampally Vijay, Abhilasha Munisingh, Vasundhara Ponnangati

**Affiliations:** 1 Cardiology, Sri Ramaswamy Memorial (SRM) Medical College Hospital and Research Center, Chennai, IND; 2 Cardiology, All India Institute of Medical Sciences, New Delhi, Delhi, IND; 3 Cardiology, Trilife Hospital, Bengaluru, IND

**Keywords:** hypokalemia related medical emergencies, hyperkalemia, digibind, yellow oleander toxicity, digoxin toxicity treatment, digoxin toxicity, oleander intoxication

## Abstract

Oleander is a prevalent tropical plant used in many parts of India for deliberate self-harm. The active ingredients act in a mechanism similar to cardiac glycosides; hence, the toxicological profile is similar to digoxin toxicity. Cardiac toxicity occurs in the form of a heart block with concomitant ventricular arrhythmia. Identifying the distinct electrocardiographic pattern for early diagnosis and initiating emergency management is imperative. Here, we present two such interesting cases of oleander intoxication, one with Nerium oleander and the other with Thevetia peruviana.

## Introduction

Oleander is a common tropical plant seen in many parts of India. All plant parts, including flowers, leaves, roots, and seeds, are poisonous upon ingestion. They contain non-digoxin-toxic cardiac glycosides like oleandrin, nerin, thevetin, oleandrin, and other unidentified substances [1,2]. It is one of the forms of common toxicological emergencies seen in certain parts of India. The clinical symptoms and toxicological profile closely mimic digoxin toxicity. Late cardiac involvement is an independent predictor of mortality in oleander poisoning. It occurs in the form of a heart block with concomitant ventricular arrhythmia. Identifying the distinct electrocardiographic pattern for early diagnosis and initiating emergency management is imperative [3]. Here, we present two interesting cases of oleander intoxication, the first likely due to the ingestion of Thevetia peruviana and the second possibly due to the ingestion of Nerium oleander.

## Case presentation

Case 1

**Figure 1:**
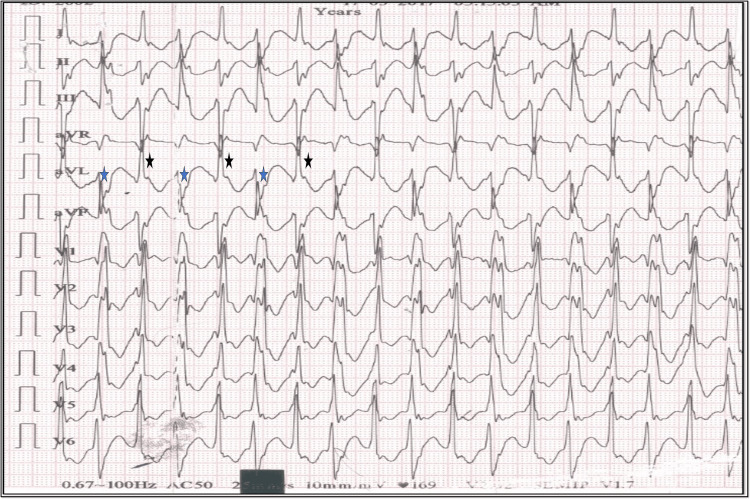
The 12-lead electrocardiogram on admission (case 1) The 12-lead electrocardiogram shows wide QRS complexes with alternating QRS axis- RBBB- blue star, LBBB-black star;  suggestive of bidirectional ventricular tachycardia.

**Figure 2:**
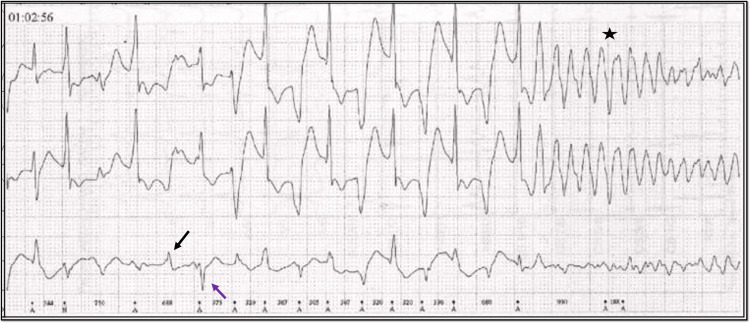
Electrocardiogram in the monitor during the cardiovascular collapse Monitor electrocardiogram showing, bidirectional ventricular tachycardia (black and blue arrow) terminating as ventricular fibrillation (black star).

A 24-year-old adult male with no prior comorbidities was presented to the emergency room (ER) with abdominal pain, vomiting, palpitations, and visual disturbances for the past six hours and worsening consciousness following a family feud. A yellow ornamental flowering plant was found in his room next to his bed. The attendees who brought him to the hospital informed us about the alleged history of ingestion of an unknown quantity following the feud, as they found an empty glass with a concoction that appeared to be consumed. On examination, he was tired and irritable, with a heart rate of 110/min, a blood pressure of 90/60 mmHg, a respiratory rate of 26/min, and normal-sized pupils reacting to light. There were no obvious facial or skeletal dysmorphisms. The electrocardiogram on admission showed bidirectional ventricular tachycardia (VT) (Figure 1). While stabilizing the patient, he had a sudden cardiovascular collapse, and the electrocardiogram on the monitor showed bidirectional VT terminating into ventricular fibrillation (Figure 2). Urgent labs revealed serum sodium of 135 meq/L, potassium of 7.0 meq/L, bicarbonate of 24 meq/L, ionized calcium of 2.2 meq/L, and magnesium of 1.6 meq/L. A quick screening echocardiogram showed no structural heart disease.

Acute onset gastric symptoms, visual disturbance, and palpitations following a family feud are suspicious of toxin ingestion. The electrocardiogram on presentation shows a wide complex tachycardia with changing QRS morphology alternating beat to beat (RBBB and LAFB alternative with RBBB and LPFB morphology), with complete atrioventricular (AV) dissociation suggestive of bidirectional ventricular tachycardia (Figure 1) which degenerated into to ventricular fibrillation (Figure 2) and caused the cardiovascular collapse. He immediately received defibrillation, amiodarone 150 mg Bolus, 10 % 10 ml calcium gluconate slow intravenous injection, and insulin dextrose infusion for correction of serum potassium. However, the patient succumbed before procurement of Digibind® (digoxin-specific antibody; GlaxoSmithKline, UK) due to refractory ventricular arrhythmia. As the patient succumbed quickly to the disease following ventricular tachycardia terminating into ventricular fibrillation (VF), there was no recovery ECG. Bidirectional ventricular tachycardia (BVT) is a rare arrhythmia with very few differentials namely, digoxin toxicity, catecholaminergic polymorphic ventricular tachycardia (CPVT), Anderson Tawil syndrome (ATS), and myocarditis. Anderson Tawil syndrome is characteristically associated with periodic paralysis and facial dysmorphism. There was no preceding febrile illness to suggest myocarditis. Catecholaminergic polymorphic VT (CPVT) is typically brought on by exertion and is generally not found at rest [1].

Case 2

**Figure 3:**
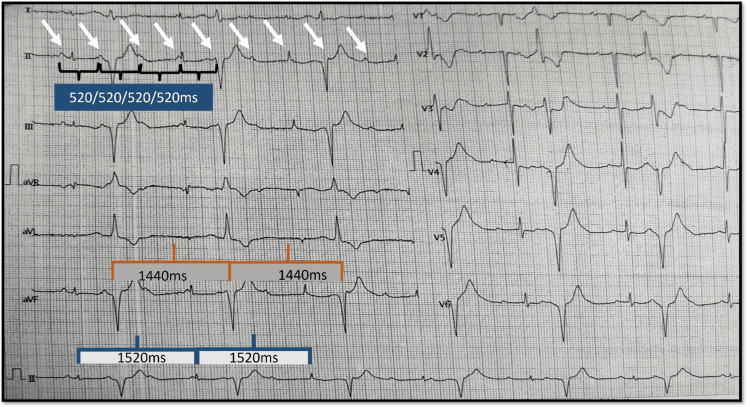
The 12-lead electrocardiogram on admission (case 2) A 12-lead electrocardiogram shows no relation between p waves and QRS complexes. The white arrows point to the series of P waves, some of which are marching through and distorting the QRS. Two QRS complex morphologies are noted. A narrow QRS complex with 100 ms duration at 1520 ms cycle length and another wide QRS complex of 130 ms at a cycle length of 1440 ms.

**Figure 4:**
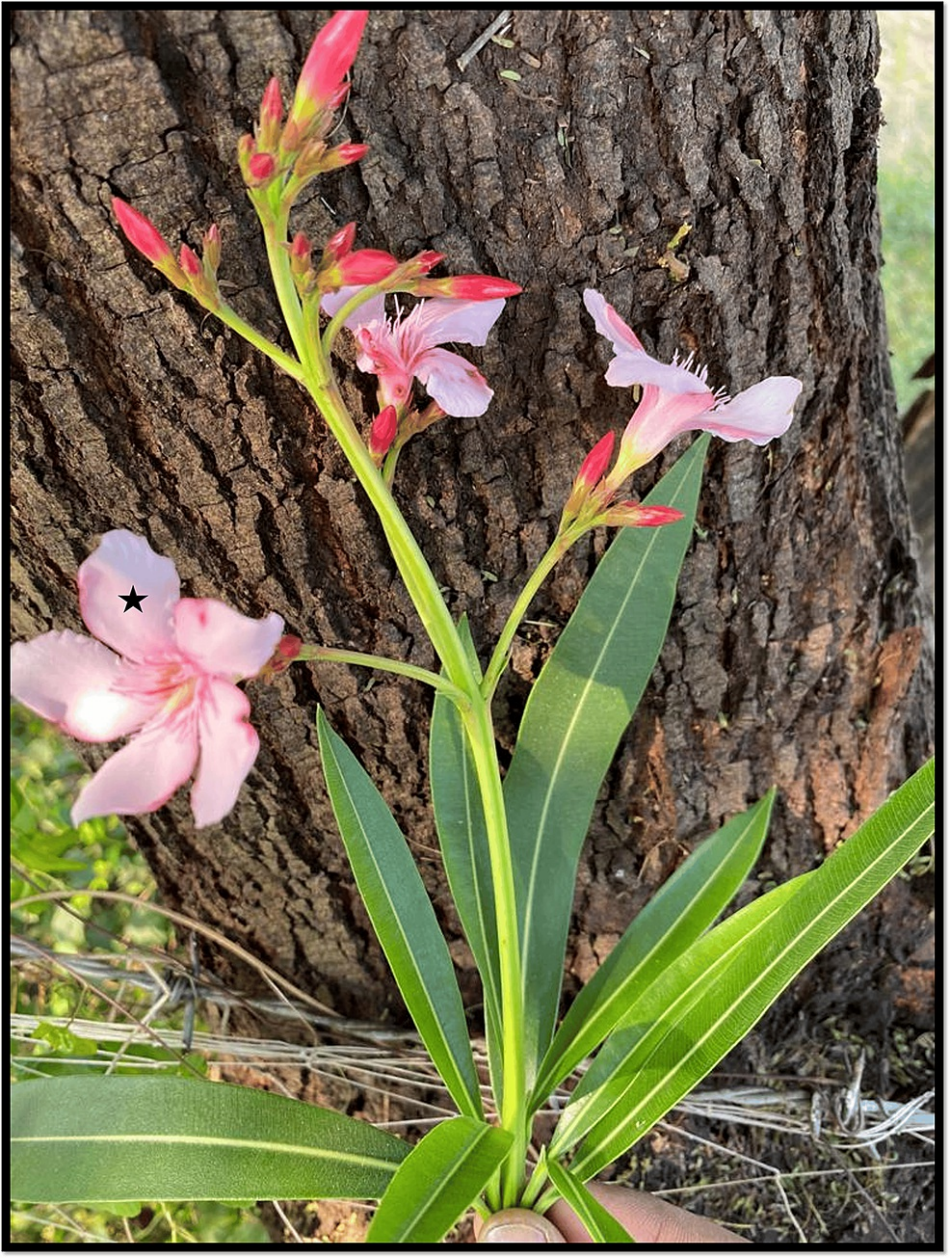
Flowers grown in the garden of case 2 Oleander (Nerium oleander) flower (black star) grown in her garden.

A 42-year-old woman, on treatment for depression with doxepin 75 mg once at bedtime, was presented to the emergency department with giddiness, presyncope, vomiting, and abdominal pain for 10 hours duration. On presentation, her blood pressure was 130/60 mm hg, and her pulse rate was 40 beats per minute and irregular. Physical examination revealed variable first heart sounds and elevated neck veins with irregular cannon waves in her jugular venous pressure (JVP). The patient's doxepin strip was checked and there was no evidence to suggest excess medication usage. Initial laboratory tests revealed hemoglobin of 12 g/dl, estimated glomerular filtration rate (eGFR) of 65ml/min/m^2^ (n <60 ml/min/m^2^), serum potassium of 3.0 mEq/L (3.5 to 5.2 mEq/L), serum magnesium of 1.7 mg/dl (1.7 to 2.2 mg/dL). The 12-lead electrocardiogram is shown below (Figure 3). On probing history further, it was found that she recently started growing new flowers in her garden (Figure 4). She was later admitted after stabilization for consumption of a concoction of approximately four flowers, stems, and seeds for deliberate self-harm.

**Figure 5:**
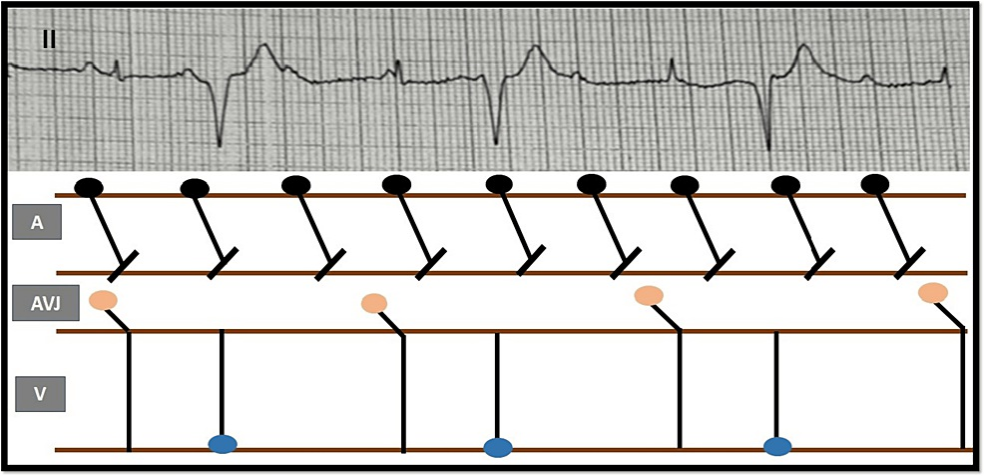
Inset of lead II above with step ladder diagram below Step ladder diagram explaining the sequences of events of atrial activity (black dot), junctional rhythm (orange dot), and ventricular ectopic (Blue dot). The step ladder image is the author's own creation.

The 12-lead ECG shows sinus rhythm with a P wave cycle length of 520 ms marching through the QRS complex suggesting complete AV dissociation. There are two morphologies of the QRS complex in a bigeminal fashion; a narrow complex QRS which is likely the junctional escape rhythm that occurs at a longer cycle length of 1520 ms, and a relatively wide QRS complex with RBBB left axis, which is a ventricular ectopic occurring at a cycle length of 1440 ms (Figure 3). Interestingly, the junctional rhythm is not inhibited by the ectopic, likely due to an entrance block in the junctional foci as demonstrated in the step ladder diagram (Figure 5).

This pattern of increased automaticity (ventricular bigeminy, and junctional rhythm) with concomitant conduction delay (AV-block) is an ECG hallmark of digoxin toxicity. The clinical picture of a middle-aged woman with a history of depression, presenting to the ER with gastrointestinal and cardiac symptoms, should raise a high index of suspicion for acute intoxication with oleander, which she was growing in her garden. The toxicological profile of oleander intoxication closely mimics digoxin toxicity. Hypokalemia occurs due to recurrent vomiting and can potentiate cardiac toxicity further. She stabilized with gastric decontamination with multi-dose activated charcoal (MDAC), intravenous slow infusion (10 meq/hour) of potassium chloride, and serial ECG monitoring. She did not require any temporary pacing after correction of hypokalemia. Appropriate psychiatric counseling was initiated, and the patient was immediately transferred to another facility with inpatient psychiatric facilities for treatment of major depression with suicidal intent. There was no complication of the intoxication or the treatment, however, no further ECG was available for further analysis.

## Discussion

One of the most common toxicological emergencies encountered in rural India is oleander intoxication. The two common types of oleanders encountered in clinical practice are Nerium oleander and Thevetia peruviana (yellow oleander). Animal studies indicate that the fatal dose of oleander intoxication is 8-10 seeds, 15-20 leaves, or 15 g of root [2,3]. At times, the patient and relatives may conceal the history of ingestion. There may be various reasons to conceal the history, as they may be afraid of the ensuing police investigations or loss of job, livelihood, or continued depression in the event of survival. Still, it is prudent for the ER physician to identify the pattern of GI symptoms, altered mental status due to cerebral hypoperfusion, and cardiac arrhythmias as patterns that should raise suspicion of toxin ingestion. The plant toxins and cardiac glycosides act on the same receptors; hence, digoxin immunoassay cross-reactivity may be used for diagnostic purposes. However, there is an inconsistent correlation between serum assays and disease severity.

Management of oleander poisoning is essentially supportive and involves early diagnosis, gastric decontamination when presenting within an hour of ingestion, Digibind®, and a temporary pacemaker in selected cases. Correcting electrolyte imbalances plays a crucial role in therapy. Aggressive fluid resuscitation is important, along with the correction of dyselectrolytemia. Hypokalemia may be caused by recurrent vomiting and can precipitate toxicity. On the contrary, hyperkalemia is a poor prognostic marker, and a potassium value of more than 5.5 meq/L is one of the indications for the administration of Digibind®, requiring urgent correction of potassium levels [4,5]. 

Multi-dose activated charcoal (MDAC) is an important therapy in the initial hours of ingestion as it helps not only in preventing absorption but also prevents enterohepatic recycling in cases of cardiac glycosides/mimetics. The prescribed doses in trials are 50 grams every six hours, but the dose may vary with each case according to the treating physician. Evidence regarding the use of MDAC is controversial, with certain studies showing a mortality benefit with the prevention of life-threatening arrhythmias and others showing no mortality benefit. 

Digoxin-specific antibodies (such as Digifab®) are not commonly prescribed and are indicated only in special cases of life-threatening arrhythmia. They have shown mortality benefits by reversing cardiac arrhythmias. However, its cost and nonavailability are its major limitations. The usual dose prescribed for an unknown ingestion quantity is approximately 20 vials (1200 mg). Certain cases of persistent bradycardia may require temporary pacing support [6].

## Conclusions

Oleander (Nerium oleander and Thevetia peruviana) has many cardiac glycosides, which act on the sodium-potassium ATP (Na-K ATPase) pump similar to digoxin. Ingestion of any part of the plant is toxic and can affect the cardiac myocytes and the autonomic nervous system. The main symptoms of intoxication are nausea, vomiting, abdominal pain, and ventricular arrhythmia with concomitant atrioventricular blocks. Treatment includes early diagnosis, supportive care, correction of electrolyte imbalances, and Digibind® in selected cases. Both cases highlight the spectrum of possible ECG changes seen in oleander intoxication, its identification, and its management.
